# Potential Distribution of the Australian Native *Chloris truncata* Based on Modelling Both the Successful and Failed Global Introductions

**DOI:** 10.1371/journal.pone.0042140

**Published:** 2012-07-27

**Authors:** Pippa J. Michael, Paul B. Yeoh, John K. Scott

**Affiliations:** 1 Department of Environment and Agriculture, School of Science, Curtin University, Perth, Western Australia, Australia; 2 Climate Adaptation Flagship, CSIRO Ecosystem Sciences, Perth, Western Australia, Australia; University Copenhagen, Denmark

## Abstract

Our aim was to model the current and future potential global distribution of *Chloris truncata* (windmill grass) based on the plant's biology, soil requirements and colonisation success. The growth response of *C. truncata* to constant temperatures and soil moisture levels were measured and estimated respectively, to develop parameters for a CLIMEX bioclimatic model of potential distribution. The native distribution in eastern Australia and naturalised distribution in Western Australia was also used to inform the model. Associations with soil types were assessed within the suitable bioclimatic region in Australia. The global projection of the model was tested against the distribution of soil types and the known successful and failed global introductions. The verified model was then projected to future conditions due to climate change. Optimal temperature for plant development was 28°C and the plant required 970 degree-days above a threshold of 10°C. Early collection records indicate that the species is native to Queensland, New South Wales and Victoria. The plant has been introduced elsewhere in Australia and throughout the world as a wool contaminant and as a potential pasture species, but some of the recorded establishments have failed to persist. The CLIMEX model projected to the world reflected effectively both the successful and failed distributions. The inclusion of soil associations improved the explanation of the observed distribution in Australia, but did not improve the ability to determine the potential distribution elsewhere, due to lack of similarity of soil types between continents. The addition of a climate change projection showed decreased suitability for this species in Australia, but increased suitability for other parts of the world, including regions where the plant previously failed to establish.

## Introduction

Projection of a species' potential distribution in areas where they are not currently found or invading is critical to weed or pest quarantine, eradication, containment and management strategies. These projections are also critical for developing adaptation strategies in anticipation of plant responses and altered community dynamics due to climate change. Usually the potential distribution is inferred from the climate associated with the current distribution (climate matching or correlative models) or from models of the species response to climate parameters (niche or mechanistic models). It is often assumed in discriminatory models that the absence of a species from a location implies that the climate at that location is unsuitable; something that may not be the case given that species invasion can be limited by biotic (e.g. biotic resistance, propagule pressure or lack of mutualisms) and other abiotic factors [1], [2]. It is also very rare that the absence of a species is truly an indication of unsuitable climate based on known introductions that have failed [3]. This is because almost without exception data on failed introductions are not available. In this paper we examine the history of successful and failed establishment of *Chloris truncata* R.Br. (Poaceae), commonly known as windmill grass. This historical record, along with information on the plant's response to temperature, soil moisture and soil type was used to develop a bioclimatic model of the potential distribution that reflects well the success and failure of establishment.

*Chloris truncata* was widely dispersed since the 18th century as contaminant of Australian wool [4], [5]. The waste from wool scouring factories located across the northern hemisphere led to numerous introductions of *C. truncata*, of which not all have survived. Proposed use as a pasture species meant further introductions including those by the prolific seed disperser, Ferdinand von Mueller, who sent *C. truncata* seeds to the French embassy in Australia in 1888 [6] (presumably for eventual introduction into Algeria). As recently as the 1970s, introductions for use as a pasture plant were made to experimental field stations in USA. Currently *C. truncata* can be obtained as an ornamental grass species via the internet. This well-documented history of mixed success of dispersal and establishment provides a rare opportunity to examine a model of a species distribution, not only to regions known to be suitable, but also to where it is known to be unsuitable.

*Chloris truncata* is a short-lived summer-active stoloniferous grass [7]. It is widely established throughout temperate regions of Australia except Tasmania 8,9. Typically *C. truncata* is a perennial species in Australia, but in south-western Australia it germinates and grows during summer becoming dormant or dying in autumn [10]. As with other perennial native grasses, there has been widespread interest in the evaluation of *C. truncata* as a component of native pastures in rangelands and temperate regions of Australia [7], [10], [11], [12], [13]. It is already considered a valuable species for controlling erosion and rehabilitating native and roadside areas [7], [14], [15], but is otherwise regarded as a weed of increasing importance to cropping in no-till agriculture.

Few native Australian species have the ability to establish in disturbed ecosystems that have regular cultivation, fertiliser inputs, ruminant grazing or crop competition [16], [17]. This includes *C. truncata* which has become a significant weed of agricultural systems, prompting the development a national strategic response in Australia [18]. It is a coloniser of bare eroded soils and disturbed areas [15] and native pastures are less productive when dominated by *C. truncata* [19]. It has also been suspected of causing photosensitisation in lambs and dermatitis in humans [15]. The plant acts as a “green bridge” over summer for diseases such as barley yellow dwarf virus, and for aphids that are disease vectors [20]. It is also an important host for the common armyworm, *Mythimna convecta* (Walker) (Noctuidae), a major pest of cereals and pastures [21]. Furthermore, when produced in high quantities, seed heads can accumulate along fences and buildings when blown by wind, causing a fire hazard [22].

Our aim was to build a model that captured both the presence and true known absences of an invasive plant species. To do this we developed a distribution model for the invasive grass *C. truncata* using the mechanistic niche model CLIMEX and methods outlined in previous studies [17], [23]. CLIMEX models the possible response of a species to climate based on geographical distribution, biology and seasonal phenology [24], [25]. This model was then projected to regions of the world using current climate and projected with a future climate scenario to account for climate change. We added to this analysis a possible response of *C. truncata* to edaphic factors in an attempt to bring greater precision to the projected distribution.

## Materials and Methods

### Growth experiments

Seed heads from mature *C. truncata* plants were harvested on 13th November 2008 from roadside edges and railway lines within the Western Australian Department of Agriculture & Food's (DAFWA) research field station in Merredin, Western Australia (31°29′35.65″S, 118°13′29.79″E). Seeds were separated from the chaff and stored (∼20°C) in a paper bag at CSIRO's laboratory in Floreat, WA (31°56′56.48″S, 115°47′25.22″E) until required. Only filled seeds with a black seed coat were retained; those with damaged or lighter coloured seed coats were discarded.

On 23rd October 2009, two seeds per cell were planted into 14 Rite Gro Kwik Pot 48 cell trays containing approximately 50 ml per cell (35×42×50 mm) of a coco peat based University of California potting mix [26]. Liquid fertiliser (Yates Thrive; N∶P∶K-27∶5.5∶9) was applied initially, then monthly at a rate of 8 mg/4.5 L. By 6th November 2009, most cells contained two emerged seedlings, which were randomly thinned to one seedling per cell. If necessary, empty cells were replaced with a cell containing a single seedling. A total of 60 seedlings (one complete tray plus an additional 12 cells) per treatment were then placed into Lindner and May environmental chambers at constant temperatures of 7, 11, 16, 19, 24, 28, 36 and 39°C (14/10 h light/dark, ∼50 µE sec^−1^ m^−2^). A further 40 plants were also placed in a glasshouse in order to determine growth under glasshouse light conditions (average temperature over the whole experimental period 24°C).

Plant size was estimated at the beginning of the experiment and at approximately monthly intervals. Live leaves were counted and a calliper or ruler used to measure the average length and width (in mm). Average leaf area was estimated by average leaf width x average leaf length ×0.8, the latter value a correction factor based on the shape of the leaves. Daily growth rates for the plants were determined by changes in total leaf area (number of leaves x average leaf area) over the month. Plants were included in the growth rate calculation if they were alive at the time of measurement, and given a value of 0% growth in the month they died. For each individual we used the longest possible period of growth to estimate its whole of life growth rate.

The experiment was terminated when several plants in the glasshouse had set seed (approximately two months after germination). Thus, in this study plant growth and development represents the full period from young seedlings to mature plants. Over a one week period, harvested plants were measured, washed to remove any soil and oven dried in paper bags to calculate dry weights. Growth rates were expressed per day to allow for these variations in time.

### Distribution records for Chloris truncata

Information on the current distribution of *C. truncata* was obtained from a wide range of literature sources [4], [5], [7], [10], [14], [19], [21], [27], [28], [29], [30], [31], [32], [33], [34], [35], [36], [37], [38], [39], [40], [41], [42] and online databases (GBIF [43], Australian Virtual Herbarium (AVH) [44], TROPICOS [45], Germplasm Resources Information Network (GRIN) [46], South African National Biodiversity Institute (SANBI) [47], Consortium of California Herbaria [48], New Zealand Virtual Herbarium [49], Bernice Pauahi Bishop Museum [50], National Museum of Natural History [51]) using the currently accepted name of the species and any other species level synonyms as listed in the Australian Plant Name Index [52]. Following data proofing, there were 1237 valid and 28 invalid records in Australia, and 138 valid and 25 invalid records for the rest of the world. Invalid records included duplicates, poor data or cultivated records.

Distribution records were depicted in two ways. If the exact location of the species was known (i.e. specific co-ordinates), then it was indicated on the map as a dot. If only a region was known, then it was indicated on the map according to Brummitt's “World Geographical Scheme for Recording Plant Distributions, Plant Taxonomic Database Standards No. 2” (Level 4, basic recording units) [53]. This system was developed by the International Working Group on Taxonomic Databases (TDWG) in response to the needs of botanists wanting biologically-based regions to record species distributions.

### The CLIMEX model and scenarios

A parameter set containing five meteorological variables, average minimum monthly temperature (Tmin), average maximum monthly temperature (Tmax), average monthly precipitation (Ptotal) and relative humidity at 09:00 h (H09:00) and 15:00 h (H15:00), was used to define weekly and annual indices that determine the species response to temperature and soil moisture. CLIMEX calculates an annual growth index (GI) based on the growth of a species under favourable conditions of temperature, moisture and light. Stress indices (cold, hot, wet and dry) and their interactions may also be added to the model to indicate species restriction during unfavourable conditions. The Growth and Stress indices are combined to create the Ecoclimatic Index (EI), an annual measure of the favourableness of a particular location for the species. Further details of the methodology are discussed in previous studies [2], [23].

The temperature indices and degree days used to inform the CLIMEX parameters were determined from laboratory and glasshouse trials as described earlier, with lower temperature threshold for growth (DV0) set at 10°C, lower optimal temperature threshold for growth (DV1) at 26°C, upper optimal temperature threshold for growth (DV2) at 34°C, and upper temperature threshold for growth (DV3) at 36°C. Degree days per generation were determined by the minimum degree-days above DV0 necessary for flowering. Moisture parameters were set to reflect the phenology of a species that grows during the extremely dry summer period in Western Australia, with a much reduced lower soil moisture threshold (SM0 = 0.055) and lower optimal soil threshold (SM1 = 0.1). The heat stress and hot-wet stress parameters were informed by the absence of the species in non-tropical and wet tropical areas of northern Australia, respectively.

As the exact boundary between native and introduced records in Australia was unknown (except for Western Australia which is clearly introduced), all Australian distribution records were considered in the iterative process used to develop the CLIMEX model. The model was then projected to the rest of the world, with global records and Brummitt's regions (established and failed) both used to assess the model.

The CliMond 10′ gridded world climate dataset [54], was used for both projected current climate (recent historical data centred on 1975) and future climate change scenario models. For a future climate scenario, the CSIRO-Mk3.0 global climate model projected to 2070 was chosen, a time considered to provide a sufficient period to allow a different distribution for a short-lived and readily dispersed species such as *Chloris truncata* to develop. The climate change scenario for 2070 was based on the IPCC emissions scenarios (the SRES scenarios or the Special Report on Emissions Scenarios) [55]. For this study we chose to work with the A1B scenario [56], which describes a future of very rapid economic growth, global populations that will peak mid-century and declines thereafter and balanced for future technological changes in fossil intensive and non-fossil energy sources. It provides a set of near mid-range values for global warming. The observed global carbon dioxide emissions during the 2000–2006 period are in line with, but above the IPCC's A1B emission scenario [57].

### Association with soil types

Associations between Australian soil types and the distribution of *C. truncata* were determined by overlaying the distribution records and soil types as given in the online “Digital Atlas of Australian Soils” [58]. Although this soil classification system is currently the best available data for Australia, it is based on the Australian situation and not readily transferred to a wider global context. EI values greater than zero were used to determine the area of Australia included in the analysis as this defined the potential distribution in the broadest sense and ensured the exclusion of soils in regions not suitable for the growth of *C. truncata*.

To enable a worldwide comparison, the same procedure was used to determine associations within Australia between the distribution and soil types based on a world classification system, the FAO-UNESCO online “World Soils Map” [59]. We then used the global database to identify suitable soil types in climatically suitable regions (as determined by the world CLIMEX projection).

### GIS methods and statistical techniques

We used ESRI ArcView Version 9.3 to generate the maps for this study. A global fishnet provided with the CliMond dataset ([54]; grid polygon shape file) at a grid size of 10′ was used to visualise the CLIMEX output. A chi-squared test was used to test the model projection for statistical significance as described in a previous study [23].

### Ethics statement

All necessary permits were obtained for the described field studies. We obtained permission from the Department of Agriculture Western Australia to collect *Chloris truncata* seeds from their Merredin field station. This was the only location used.

## Results

### Current distribution in Australia

**Figure 1:**
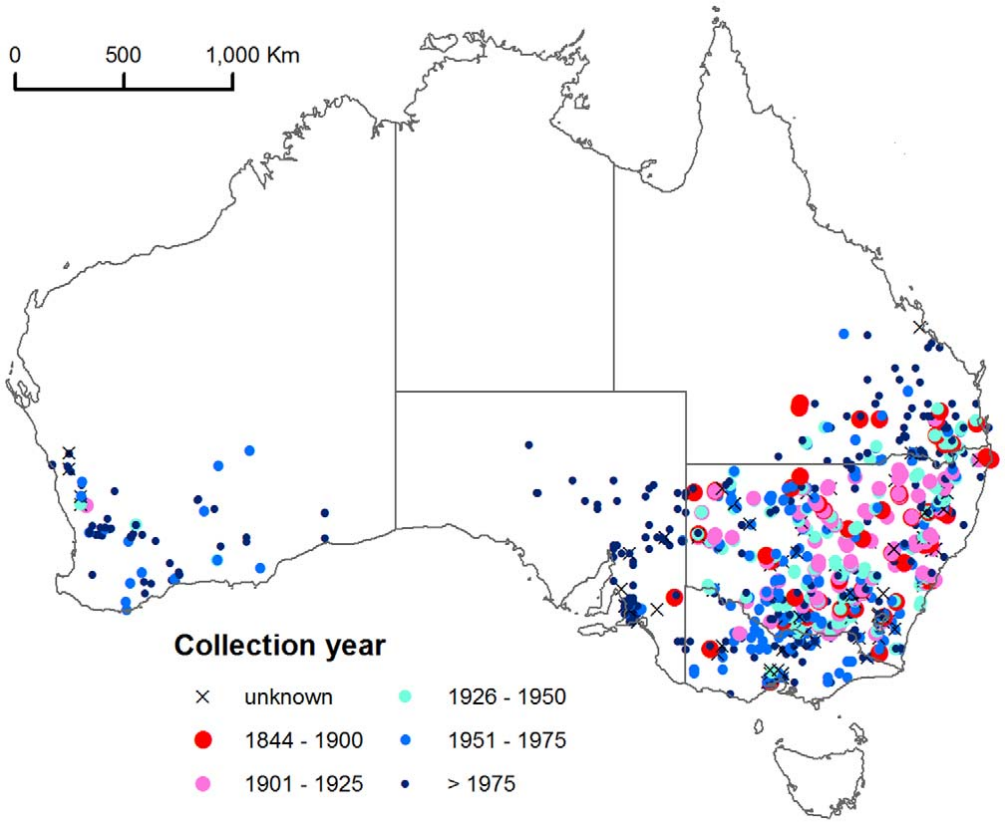
Distribution of *Chloris truncata* in Australia as categorised by collection period.

The 1237 distribution records for *C. truncata* in Australia showed that early records from 1844–1900 (37) are from NSW, southern Queensland, Victoria and South Australia (Fig. 1). We considered the few early records in Western Australia to be evidence of early human-mediated location records rather than reflecting lack of collecting. The early records cover both dry and temperate coastal regions. There is clear evidence for a spread westwards into Western Australia, with a possibly minor spread northwards in Queensland and westwards in South Australia, which we interpret as a range expansion. The grass is absent from Tasmania, the driest inland regions (the single NT record is not a true naturalisation; see Table 1) and tropical regions.

**Table 1:** Global records of the introduction and establishment success of *Chloris truncata*.

Country/Region	Date of first record	Means of introduction	Current status^1^
Argentina	1936	Not stated [36]	Naturalised [36]
Australia, Lord Howe Island	1962	Pasture [69]	Naturalised [9]
Australia, Northern Territory	1984	Not stated [9]	Casual alien [9]
Australia, South Australia	1890	Wool [16]	Naturalised [52]
Australia, Tasmania	1998	Pasture [61]	Extinct [61]
Australia, Western Australia	1925	Not stated [44], likely to be sheep	Naturalised [52]
Belgium	1887	Wool [33]	Casual alien [70]
Czech Republic	1958–61	Wool [71]	Extinct [4]
Fiji	1927	Not stated [41]	Not recorded as present [72], unlikely to be naturalised [73]
France^2^	1892	Wool [38]	Not recorded as present [74]
Germany	1889	Wool [38]	Not recorded as present [74]
Japan	1962	Not stated [37], [75], records found near wool importing port and processing area	Establishment not confirmed
Netherlands	1940	Wool [76]	Not recorded as present [74]
New Zealand	1877	Pasture [77]	Naturalised [64]
Niue	1965	Not stated [50]	Not present [78], incorrectly identified herbarium specimen
Philippines	1816	Not stated [79]	Not present [79], [80]
Poland	1897	Wool [38]	Not recorded as present [74]
Spain, Mainland	2003	Not stated [5]	Naturalised [42]
Spain, Canary Islands	2003	Not stated [81]	Naturalised [5]
South Africa	1901	Not stated [47]	Naturalised [63]
Sweden	1935	Wool [38]	Not recorded as present [74]
Switzerland	1926	Wool [38]	Not recorded as present [74]
Tonga	Unknown	Not stated [50]	Establishment not confirmed
United Kingdom	1915	Wool [38]	Not established [82]Casual alien [62]
USA, California	1942	Not stated [65]	Naturalised [65]
USA, Georgia	1969	Not stated [45], record appears to be from a long-term experimental research trial	Establishment not confirmed
USA, Hawaiian Islands	1904	Not stated [50]	Naturalised [35]
USA, South Carolina	1957	Wool [40]	Not recorded as present [83]

1 Definition of casual and naturalisation [84].

2 Whilst Ferdinand von Mueller sent *C. truncata* seeds to the French embassy in Australia in 1888 [6], there is no record of introduction into North Africa or France as a result nor current records of naturalisation.

### Current distribution overseas

**Figure 2:**
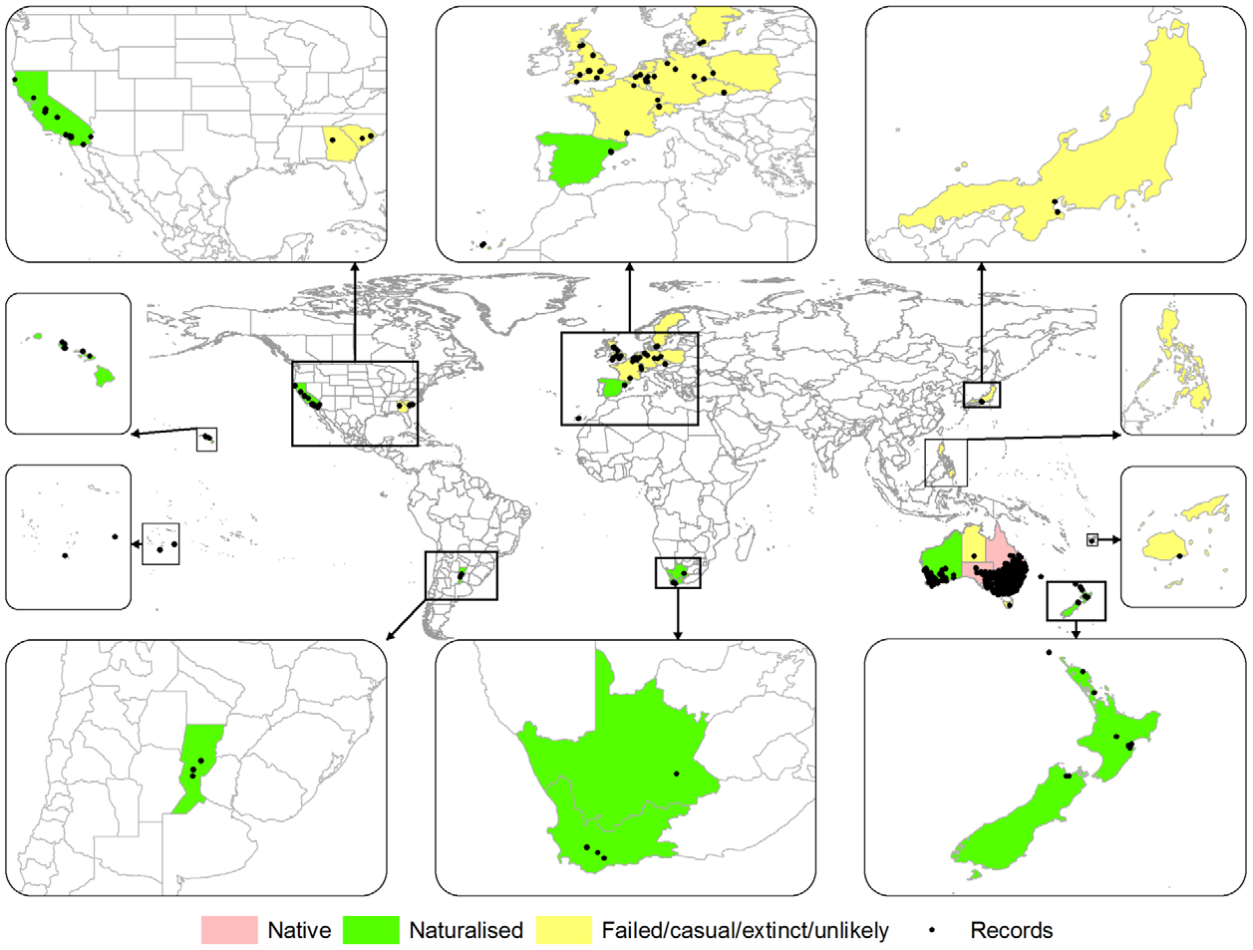
World-wide distribution of Chloris *truncata* showing established, false and failed locations.

*Chloris truncata* has been recorded in 23 regions outside of Australia (Table 1; Fig. 2), including some misidentifications and likely erroneous records. There are ten cool temperate regions of known introductions as a wool alien that have failed to persist, indicating that these regions may have marginal climatic suitability or are outside the fundamental climatic niche for this species. Eight regions outside mainland Australia, from all areas of the globe, have confirmed establishment (Table 1). These records are all from regions of warm temperate or mediterranean-type climates. Two means of introduction were identified: (i) as a contaminant in wool, and (ii) as a deliberately introduced pasture plant, although for some locations the means of introduction was not possible to confirm conclusively (e.g. Japan Table 1).

### Growth in relation to temperature

**Figure 3:**
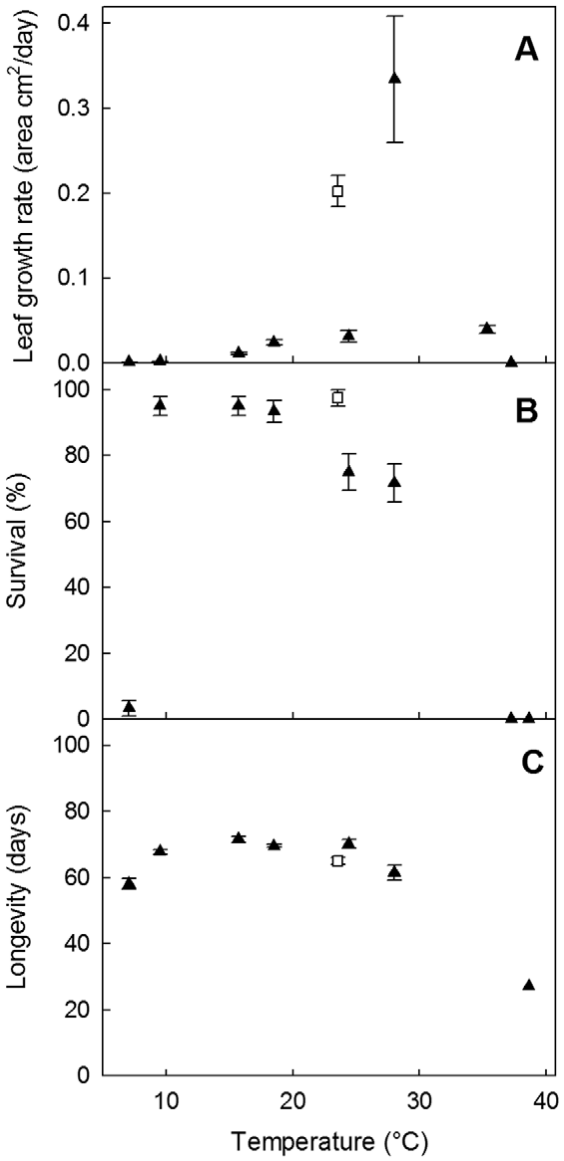
Leaf growth rate (A), plant survival (B) and plant longevity (C) (± se) of *Chloris truncata* under constant temperature (▴, n = 60) and glasshouse (□, n = 45) conditions.

The vegetative biomass production of plants growing within the environmental chambers was optimal at a constant 28°C where plants had an average dry weight of 94.0±31.4 mg (± SE, n = 45). Biomass declined rapidly with slight deviations from this temperature with plants weight averaging less than 3.8 mg in the chamber set only 5°C lower or higher. Plants growing in the glasshouse were, however, 5 times heavier than their largest counterparts growing concurrently in the environmental chambers (mean dry weights of 469.5±30.3 mg, n = 39). Even so, plants in the environmental chamber running at 28°C produced greater leaf area (cm^2^/day) than even the plants in the glasshouse (Fig. 3). The temperature range (parameters needed for the CLIMEX model) for vegetative growth was very restricted, being substantially less when lower than 15°C or higher than 35°C (Fig. 3). The 28°C chamber was also the only one in which plants started reproductive growth. Taking all aspects of measured plant growth into consideration, the temperature range for vegetative and reproductive growth was approximately 10 to 36°C (lower and upper thresholds, respectively). Within this range, there was only a narrow optimal temperature band, being higher than 26°C but lower than 34°C (Fig. 3).

For seedlings that were initially 3 days old and kept in the glasshouse for their entire life, the average time from the start of the experiment until the plants produced seed was 50±1.5 (n = 34) days. All growth experiments were concluded when the plants in the glasshouse had produced seed (2 months after emergence). Although some (12 out of 60) of the plants in the temperature chamber running at 28°C had produced reproductive stems, no plants in any of the chambers had produced seed during the experimental period. Based upon 34 individuals that produced seed in the glasshouse, the average minimum Day Degrees above a Lower Developmental Threshold value of 10°C was 970°D from emergence to the start of seed production.

### The CLIMEX model

**Figure 4:**
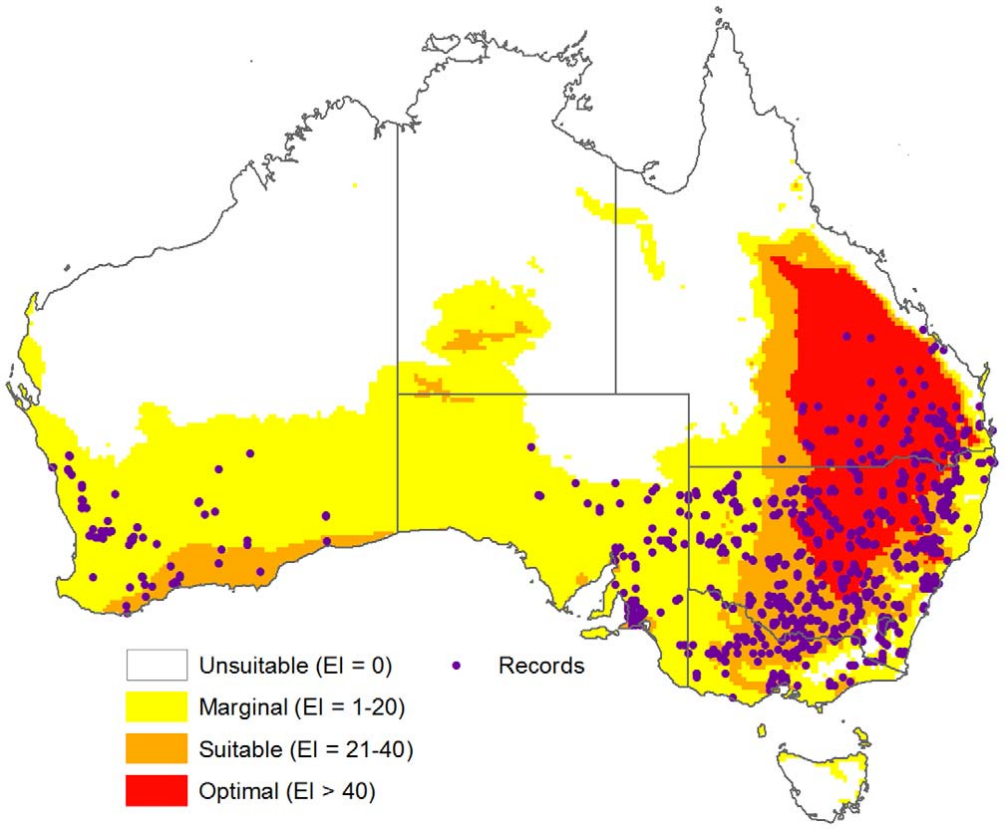
Known Australian distribution records and projected current climate suitability for *Chloris truncata*. CLIMEX climatic suitability as shown by the Ecoclimatic Index (EI) is indicated by the changing colour scale: Unsuitable (EI = 0), Marginal (EI = 1–20), Suitable (EI = 21–40), Optimal (EI>40).

The CLIMEX model (Table 2) showing current climatic suitability (Fig. 4) had high sensitivity, covering 99% of all known distribution records in Australia, and showed the absences in Tasmania, the Australian Alps, tropical and dry inland regions. It also indicated that significant regions of southern and central Australia were suitable for the species. Modelled prevalence for Australia, or proportion of the model universe estimated to be climatically suitable, was 0.5. The model projection was highly statistically significant (P<0.0001) when tested against known distribution records in Australia (Table S1).

**Table 2:** CLIMEX parameters values used for modelling the distribution of *Chloris truncata* based on the temperature requirements for development, native (Australian) distribution and phenology data.

Index	Parameter	Values	Units
Temperature	DV0 = lower threshold	10	°C
	DV1 = lower optimum temperature	26	°C
	DV2 = upper optimum temperature	34	°C
	DV3 = upper threshold	36	°C
Moisture	SM0 = lower soil moisture threshold	0.055	
	SM1 = lower optimum soil moisture	0.1	
	SM2 = upper optimum soil moisture	0.45	
	SM3 = upper soil moisture threshold	0.8	
Heat stress	TTHS = temperature threshold	36.9	°C
	THHS = heat stress accumulation rate	0.45	Week^−1^
Hot-wet stress	TTHW = Temperature threshold	27.5	°C
	MTHW = Soil moisture threshold	0.4	
	PHW = stress accumulation rate	0.085	Week^−1^
Degree days per generation	Number of degree-days above DV0 necessary to complete one generation	970	°C days

Note that parameters without units are a dimensionless index of plant available soil moisture scaled from 0 (oven dry) to 1.0 (field capacity).

When the CLIMEX model was projected globally (excluding Antarctica) it indicated a mainly Mediterranean-type climatic potential distribution for *C. truncata*, in addition to parts of eastern and southern Africa, eastern Brazil, Argentina, Paraguay and southern Bolivia, China and the USA. The model had high sensitivity (0.64) and specificity (0.84), the proportion of true absences occurring in climatically unsuitable areas, (e.g. central and northern Europe, eastern USA, Japan) (Table S1). Areas where records were inaccurate or not confirmed were unsuitable for establishment (Philippines, tropical islands). The model projection was highly statistically significant (P<0.0001) when tested against known distribution records globally and the modelled prevalence was 0.24 (0.22 excluding Australia).

### Impact of climate change

**Figure 5:**
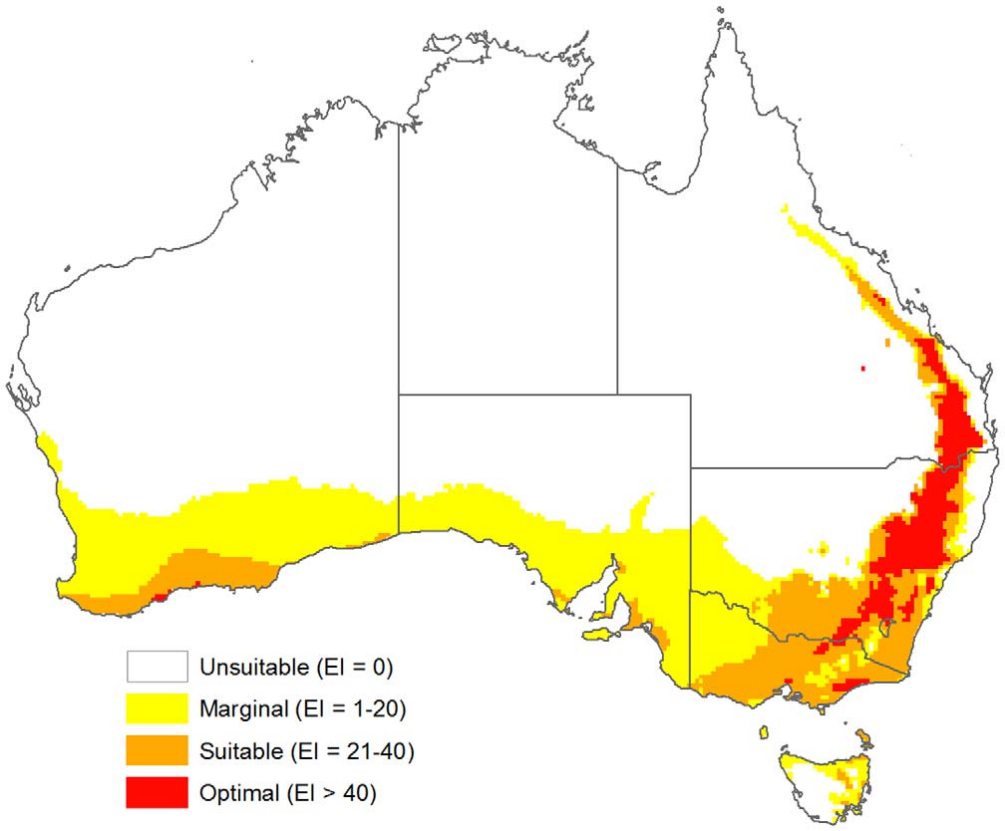
Projected future climate suitability for *Chloris truncata* as shown by the Ecoclimatic Index (EI) using CSIRO Mk3 projections for 2070 under the SRES A1B emissions scenario. CLIMEX climatic suitability as shown by the Ecoclimatic Index (EI) is indicated by the changing colour scale: Unsuitable (EI = 0), Marginal (EI = 1–20), Suitable (EI = 21–40), Optimal (EI>40).

**Figure 6:**
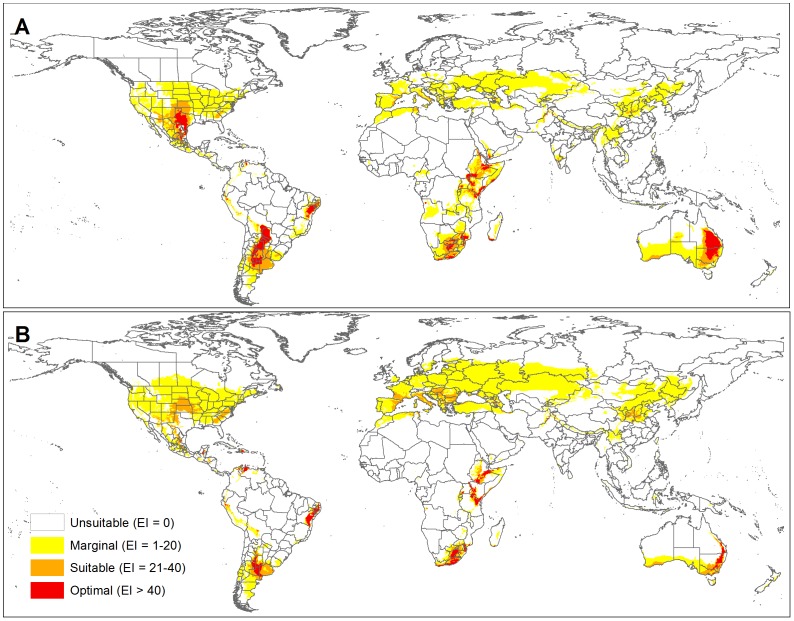
Projected world distribution of CLIMEX climatic suitability as shown by the Ecoclimatic Index (EI) is indicated by the changing colour scale: Unsuitable (EI = 0), Marginal (EI = 1–20), Suitable (EI = 21–40), Optimal (EI>40).

In Australia the projected distribution for the 2070 A1B climate scenario contracts polewards (i.e. south) and largely become confined to more coastal regions in the southern half of the continent (Fig. 5). The most favoured region climatically, contracts considerably to the south east. At a world scale *C. truncata* has a projected increase in distribution in the Mediterranean region through to southern Russia and Kazakhstan (Fig. 6). The distribution is projected to decrease in southern Africa, east Africa, southern USA and Argentina. In the northern hemisphere there is an increased distribution polewards whereas in the southern hemisphere, the decreasing distribution in climatic suitability coincides with the continental edges, although this is not the case in South America.

### Association with soil type

*Chloris truncata* was found on all soils in the Australian landscape (Table 3), but is more frequently associated with heavier soils. Of the 1218 collection localities mapped in Australia, 570 (47%) were associated with the soil landscape classifications of red duplex soil and cracking clays, despite these soils comprising 21% of the area that is climatically suitable for *C. truncata*. Conversely, few collection records (85 or 7% of total records) were associated with calcareous earths and sands (representing 31% of the climatically suitable area; Table 3). Soil associations were also evident from the FAO data for Australia (Table S2), with a very strong positive association with calcic luvidols, strong positive associations with chromic luvisols and solodic planosols, but a negative association with ferralic arenosols and calcic xerosols.

**Table 3:** Association of collection records of *Chloris truncata* with soil types in Australia [58] found within the projected area of climate suitability (EI>0).

Soil	Area (km^2^)	Number of records	Expected number of records based on area	% contribution to total X^2^
Brown duplex	43,442	13	14	0
Calcareous earths	389,302	38	124	7
Cracking clay	420,602	231	134	9
Grouped minor soils	41,391	20	13	0
Loams	520,434	72	166	7
Massive earths	770,807	234	246	0
Red duplex	382,460	339	122	48
Sands	812,695	47	259	22
Yellow duplex	436,310	224	139	6
	3,817,444	1218	1218	

X^2^ test of association = 798.3, 8 df, P<0.001. Soils with fewer than 5 observed records were combined under “Grouped minor soils” (black duplex, grey duplex, non-cracking clays, bare rock, lakes, organic & no data).

The Australian soil classification (Table 3) is unique to Australia, which means that the FAO soil classification is the only option for projecting the soil associations found in Australia to the rest of the world. However, the soils with strong associations with *C. truncata* within Australia are either of limited distribution outside of Australia, or are found in areas outside of the climatically suitable area projected by the CLIMEX model (Fig. S1).

## Discussion

Our confidence in the species distribution model (CLIMEX) benefits greatly from having data based on plant establishment failure that can be used to inform the model. It is rare to be able to include this aspect in species distribution models due to the ephemeral nature of failed introductions. However, it is fortuitous that *Chloris truncata* was a “wool alien” during a time when the recording of these species was receiving attention. The extensive and relatively abundant collection records were also vital for testing the climate model and association with soil types. It is important to note that climate alone is not the only factor in determining regions suitable for invasion, with biotic factors potentially playing a major role in preventing some introductions from becoming invasions [1]. *Chloris truncata* is becoming economically important in Australia [18] and may represent a quarantine risk elsewhere so it is timely to examine issues regarding potential distribution.

### Australian distribution

The herbarium records used in our map (Fig. 1) date from 1844 and indicate a widespread south eastern Australian origin for *Chloris truncata*, not a more interior origin as proposed previously [16]. The Type specimen was collected in 1810 without a location more precise than Port Jackson ( = New South Wales; [8]), however given the date, the collection would have been nearer to the coast than inland.

The absence in Tasmania is supported by it not being recorded among grasses of this island [60]. Also *C. truncata* has been trialled as a pasture species in Tasmania, but failed to survive and establish [61]. Both records in the Northern Territory were collected in 1984 from a watered lawn in Alice Springs, but the plant did not naturalise [9], suggesting the absence of other records in central Australia is ecologically meaningful. There are no literature records of *C. truncata* in tropical Australia.

Herbarium records clearly indicate that *C. truncata* was introduced to Western Australia and probably spread southwards from the native range into South Australia via the movement of sheep [16]. The earliest record for Western Australia is 1939 at Moora and Salmon Gums, located over 500 km apart indicating multiple introductions, possibly by the movement of sheep, seed or farm machinery from eastern Australia. Recently the species has been trialled as a pasture plant in Salmon Gums [10].

The CLIMEX model encompassed the current distribution of *C. truncata* in Australia reasonably well, including the species absence in Tasmania and tropical regions. The model indicates that climatically suitable regions exist beyond the current distribution in western South Australia, eastern and central west coastal Western Australia and southern parts of the Northern Territory. With widespread interest in the evaluation of *C. truncata* as a pasture component [7], [10], [11], [12], [13] and as a rehabilitation species [7], [14], [15], it is likely the species will become further established via deliberate introductions within agricultural systems. If these systems continue to remain predominately crop/pasture rotations rather than permanent pastures, the implications of this shift in distribution could indicate a major threat to wheatbelt farming systems in the future. However, if systems become more pasture based, one likely scenario after climate change, this species may actually turn out to be beneficial to land-owners.

### Worldwide distribution

Many of the global records of *C. truncata* have resulted from introduction as “wool-aliens” and appear in climates where the species is unlikely to have successfully naturalised and persisted. Hence in the UK it is known as a “casual” species (i.e. not persisting more than two years without re-introduction [62]), indicating that it is not established as part of the alien flora. In the past, *C. truncata* has been described as a “regular but ephemerous wool-alien” of Europe and the United States [5]. Several sources have indicated that *C. truncata* is currently extinct within European regions such as the Czech Republic [4], [5]. A study by Pysek [4] found that Australian plants introduced as wool-aliens in the Czech Republic had a very poor survival rate and were unlikely to naturalise. The study also found a correlation between the success of wool-alien plants within the UK and in Central Europe, indicating that the same species tend to be successful elsewhere in Europe. The CLIMEX model shows these regions as either being unsuitable or marginally suitable for establishment of *C. truncata*.

Nonetheless, records from Spain [5], South Africa [63], New Zealand [64], Argentina [36], California [65] and Hawaii [35] indicate *C. truncata* is well established as an invasive alien species elsewhere. These areas fall within the higher EI values of the projected distribution produced by the CLIMEX model, representing a closer match to the eco-physiological requirements experienced in the native range.

Globally the CLIMEX model predicts that regions such as southern Africa, eastern Europe and Asia are likely to become more climatically suitable for survival of the species in the future with projected climate change. Thus there is a potential for this native Australian plant to become a bigger threat internationally in the future in regions not currently at risk.

### Soil associations

An association of *C. truncata* with certain soil types was noticed in 1935 by Everist who stated that the species “favours black soil open downs, edge of red soil country” [66], and this was supported by the test of association against soil classification data (Table 3). A caveat must be raised here as there is a lack of detailed information on the variation within each polygon in the soil data used for this study [67]. Sometimes the specified (dominant) soil type may occupy only a limited area (e.g. 20%), so a test of association is, at best, a coarse measure. We found that the international scale soil maps were of limited use for identifying world regions where establishment might be favourable for *C. truncata* because the unique nature of Australian soils was still apparent. In this respect, the proposed new world soil map [68] has the potential to change this situation, but it may still be that geologically very old parts of the world, like Australia, will remain under particular soil classifications, preventing the matching of soil types. For this reason parameterising additional soil components (e.g. phosphorous levels, pH) that are known to influence plant growth and survival should be investigated, as this may enable novel suitable soil types to be putatively identified.

### Conclusions

*Chloris truncata* has had a long and unusually well documented history as a global traveller with mixed invasion success. Dispersal has occurred through a diverse range of pathways, via contaminated Australian wool transported to scouring factories on the other side of the world, to deliberate dispersal as a pasture plant, through to a current potential redistribution in the mail via the web as part of the fashion for ornamental grasses. For the most part these destinations are to climatic regions that can be assessed for invasion risk via bioclimatic models informed by information on the plant's biology such as development in relation to temperature. For this reason species distribution models are important for determining invasion potentials and are critical to pest quarantine, eradication, containment and management strategies. In contrast, the strong association with soil types in Australia could not be projected to other parts of the world due to a lack of appropriate data. These non-climatic influences on the potential distribution of invasive species are important to understand in more detail and efforts to develop alternative methods for integrating such data should continue.

## Supporting Information

Figure S1 **FAO soil types within the projected area of climatic suitability (EI>0) for** ***Chloris truncata*** **.**
(TIF)

Table S1 **Number of** ***Chloris truncata*** **records within each country.**
(DOCX)

Table S2 **Association of** ***Chloris truncata*** **collection locations and FAO soil types within the projected area of climatic suitability (EI>0) in Australia.** X^2^ test of association = 1236.5, d.f. = 24, P<0.001. Soils with fewer than 5 observed records were combined under Grouped minor soils.
(DOCX)
