# Supplementary material for: Potential Distribution of the Australian Native Chloris truncata Based on Modelling Both the Successful and Failed Global Introductions
Source: PLoS One. 2012 Jul 27;7(7):e42140. doi: 10.1371/journal.pone.0042140 (PMC3407094; doi:10.1371/journal.pone.0042140)
Supplement: Table S1 — Number of Chloris truncata records within each country. (DOCX) [file pone.0042140.s002.docx]

**Table S1.** **Number of *Chloris truncata* records within each country.**

| **Country** | **Establishment** | **Records within unsuitable area (EI = 0)** | **Records within suitable area (EI > 0)** | **Total** |
| --- | --- | --- | --- | --- |
| Australia | Established | 13 | 1224 | 1237 |
|  | Failed | 1 | 1 | 2 |
| Argentina | Established | 0 | 3 | 3 |
| Belgium | Failed | 7 | 0 | 7 |
| Czech Republic | Failed | 0 | 1 | 1 |
| Fiji | Failed | 1 | 0 | 1 |
| France | Failed | 0 | 2 | 2 |
| Germany | Failed | 6 | 2 | 8 |
| Japan | Failed | 2 | 0 | 2 |
| Netherlands | Failed | 3 | 1 | 4 |
| New Zealand | Established | 10 | 7 | 17 |
| Niue | Failed | 1 | 0 | 1 |
| Poland | Failed | 0 | 1 | 1 |
| Spain, Mainland | Established | 0 | 3 | 3 |
| Spain, Canary Is | Established | 0 | 2 | 2 |
| South Africa | Established | 0 | 9 | 9 |
| Sweden | Failed | 3 | 0 | 3 |
| Switzerland | Failed | 4 | 0 | 4 |
| Tonga | Failed | 1 | 0 | 1 |
| United Kingdom | Failed | 18 | 0 | 18 |
| USA, California | Established | 8 | 25 | 33 |
| USA, Georgia | Failed | 0 | 1 | 1 |
| USA, Hawaiian Is | Established | 11 | 2 | 13 |
| USA, South Carolina | Failed | 2 | 0 | 2 |
