# Supplementary material for: Potential Distribution of the Australian Native Chloris truncata Based on Modelling Both the Successful and Failed Global Introductions
Source: PLoS One. 2012 Jul 27;7(7):e42140. doi: 10.1371/journal.pone.0042140 (PMC3407094; doi:10.1371/journal.pone.0042140)
Supplement: Table S2 — Association of Chloris truncata collection locations and FAO soil types within the projected area of climatic suitability (EI>0) in Australia. X2 test of association = 1236.5, d.f. = 24, P<0.001. Soils with fewer than 5 observed records were combined under Grouped minor soils. (DOCX) [file pone.0042140.s003.docx]

**Table S2.** **Association of *Chloris truncata* collection locations and FAO soil types within the projected area of climatic suitability (EI > 0) in Australia.** X^2^ test of association = 1236.5, d.f.= 24, P < 0.001. Soils with fewer than 5 observed records were combined under Grouped minor soils.

| **Soil** | **Area**  **km^2^** | **Number of records** | **Expected number of records based on area** | **% contribution to total X^2^** |
| --- | --- | --- | --- | --- |
| Calcic Luvisols | 198390 | 208 | 63 | 27 |
| Calcic Xerosols | 603899 | 50 | 191 | 8 |
| Cambic Arenosols | 18157 | 5 | 6 | 0 |
| Chromic Luvisols | 111830 | 110 | 35 | 13 |
| Chromic Vertisols | 359996 | 177 | 114 | 3 |
| Distric Nitosols | 30596 | 10 | 10 | 0 |
| Dystric Regosols | 196629 | 12 | 62 | 3 |
| Eutric Cambisols | 15438 | 23 | 5 | 5 |
| Eutric Nitosols | 16053 | 7 | 5 | 0 |
| Eutric Regosols | 30903 | 5 | 10 | 0 |
| Ferralic Arenosols | 403179 | 5 | 128 | 10 |
| Ferric Luvisols | 15170 | 21 | 5 | 4 |
| Gleyic Acrisols | 7029 | 5 | 2 | 0 |
| Grouped minor soils^1^ | 239744 | 16 | 76 | 4 |
| Haplic Kastanozems | 10881 | 10 | 3 | 1 |
| Humic Podzols | 25847 | 24 | 8 | 2 |
| Lithosols | 160222 | 23 | 51 | 1 |
| Luvic Phaeozems | 17109 | 14 | 5 | 1 |
| Luvic Yermosols | 657194 | 120 | 208 | 3 |
| Orthic Acrisols | 21451 | 21 | 7 | 2 |
| Orthic Solonchaks | 53930 | 7 | 17 | 0 |
| Orthic Solonetz | 243537 | 96 | 77 | 0 |
| Pellic Vertisols | 58020 | 41 | 18 | 2 |
| Rhodic Ferralsols | 17225 | 8 | 5 | 0 |
| Solodic Planosols | 303098 | 190 | 96 | 7 |
|  | 3815526 | 1208 | 1208 |  |

^1^Humic Acrisols, Chromic Cambisols, Ferralic Cambisols, Humic Cambisols, Xanthic Ferralsols, Humic Gleysols, Haplic Phaeozems, Calcic Kastanozems, Albic Luvisols, Orthic Luvisols, Dystric Histosols, Orthic Podzols, Albic Arenosols, Calcaric Regosols, Eutric Planosols, Humic Planosols, Haplic Xerosols, Haplic Yermosols and

Takyric Solonch
